# Sustaining blended and online learning during the normal and new normal conditions in a Saudi higher education institution: health science students' perspectives

**DOI:** 10.1016/j.heliyon.2022.e10898

**Published:** 2022-10-04

**Authors:** Nouf Al-Kahtani, Abdullah Almurayh, Arun Vijay Subbarayalu, Tunny Sebastian, Hend Alkahtani, Duaa Aljabri

**Affiliations:** a Health Information Management and Technology Department, College of Public Health, Imam Abdulrahman Bin Faisal University, Dammam, Saudi Arabia; b Department of Educational Technologies, College of Education, Imam Abdulrahman Bin Faisal University, Dammam, Saudi Arabia; c Quality Assurance Department, Deanship of Quality and Academic Accreditation, Imam Abdulrahman Bin Faisal University, Dammam 31441, P. O. Box 1982, Saudi Arabia; d Department of Clinical Nutrition, College of Applied Medical Sciences, Imam Abdulrahman Bin Faisal University, Dammam, Saudi Arabia; e Department of Information Systems, College of Computer and Information Sciences, Princess Nourah bint Abdulrahman University, Saudi Arabia; f Department of Health Information Management and Technology, College of Public Health, Imam Abdulrahman Bin Faisal University, Dammam, Saudi Arabia

**Keywords:** Blended learning, COVID-19, Health science students, Online learning, Perception

## Abstract

**Background:**

Higher education institutions (HEIs) shifted from in-person attendance to blended and online learning due to the COVID-19 lockdowns.

**Objective:**

This study investigated the students' perception of satisfaction, convenience, engagement, and learning towards blended and online courses conducted before, during, and after the COVID-19 lockdowns.

**Methods:**

A longitudinal study design was adopted to examine the students' perception of online and blended learning courses before, during, and after the COVID-19 lockdowns. The subjects consist of Health science students (N = 130) belonging to two different colleges of a public university and the study period includes three academic years (i.e., six semesters) (2018–2021). A survey tool was developed to collect data from students studying the computer fundamentals course through blended and online learning modes from 2018 to 2021.

**Results:**

Over 95% of students have been satisfied with the course offered through various blended learning formats since 2018. The blended 0.50-course format is the most preferred one for the students; however, the Blended 0.75-course format is highly rated by the students regarding their satisfaction and engagement than other learning formats during the COVID-19 lockdowns. Following adaption after COVID-19, the students reported a high perception of learning towards the course when delivered through 100% online learning mode.

**Conclusion:**

Students' achievement is significantly associated with the learning modes, exam modes, and various student batches. The blended 0.75-course format group shows a higher achievement than the other three-course format groups. Likewise, those students who appeared in their exams online showed higher achievement than those who appeared physically. Further, the students felt equally convenient with Blended 0.75 and 100% online learning course formats. These findings would also help HEIs choose appropriate learning and examination modes while designing courses.

## 1 Introduction

Presently, information and communication technology have significantly affected nearly every part of modern human life, impacting all industries, including education, commerce, business, and telecommunication, to news and entertainment. To a great deal, the internet has introduced innovative interactions that are recently becoming a fundamental and powerful tool or device for learning, training, and teaching in numerous institutions worldwide. In higher education institutions (HEIs), a learning management system, is an online platform to manage educational materials, assignments, communications, and course instructions (Abu Shawar, 2009). It supports blended and online learning (Kabassi et al., 2016). Typically, the blended learning environment combines e-learning benefits with other advantages of traditional teaching strategies like face-to-face contact (Finn and Bucceri, 2006). An earlier researcher also defined blended learning as integrating face-to-face and computer-mediated teaching. This definition is the concept of combining two modes of course delivery (Graham, 2006). In blended learning, the students and faculty members are well versed traditional and online methods and experience face-to-face interaction in virtual space (Lalima and Dangwal, 2017). Furthermore, blended learning might increase learning results, boost motivation for students, and is a practical approach to achieving learning goals. Hybrid education also costs less for training and may improve the learning experience of pupils (Moore et al., 2011). On the other hand, online learning is essentially the use of technology for information dissemination (Tajik and Vahedi, 2021). It is termed as learning experiences in synchronous or asynchronous environments using mobile phones, laptops, etc., with internet access. It is an instrument that makes the teaching-learning process more flexible, innovative, and student-centered (Dhawan, 2020). Due to technological innovation, several HEIs have adopted online learning strategies for multiple reasons. Those reasons include providing flexibility and convenience to all students, better use of space and, more specifically, classroom space, and more so if students are assured of achievement through physical classes (Owston and York, 2018). Additionally, they offer to learn to develop new programs or degrees capable of serving other parts of the world. Online education helps instructors and students with technology to enable time, privacy, and independent study. This convenience and flexibility allow people to manage jobs, education, and families (Nortvig et al., 2018). Besides, assessing the students' satisfaction is significant to HEIs to aid them in identifying their strengths and areas for improvement (Li et al., 2016). Accordingly, the students' satisfaction is critical in measuring the quality of blended learning (Naaj et al., 2012). High student satisfaction levels are essential elements of blended learning value (Naaj et al., 2012). Further, determining the student’s satisfaction with online learning is vital to effectively encouraging educational processes for students, instructors, and HEIs (Elshami et al., 2021).

Moreover, HEIs worldwide support blended and online learning in normal conditions (i.e., before COVID-19) (Bashir et al., 2021; Weerathunga et al., 2021). However, the entire world experienced the lockdown due to the COVID-19 pandemic, and such lockdown pushed all HEIs to shift to online mode instead of an in-person class environment (Wani, 2020). Accordingly, all Saudi HEIs also turned to online learning to control the COVID-19 outbreak (Aziz Ansari et al., 2021). While reviewing the past literature, global researchers have discussed the students' perceptions of blended and online learning during different periods, i.e., before or during, or after the COVID-19 lockdowns across the globe (Aji et al., 2020; Akuratiya and Meddage, 2020; Almahasees et al., 2021; Atwa et al., 2022; Bali and Liu, 2018; Bashir et al., 2021; Gyamfi and Gyaase, 2015; Law et al., 2019; Muthuprasad et al., 2021; Ntim et al., 2021; Nurmasitah et al., 2019). Especially in the Saudi Arabian context, various studies have revealed the students' perceptions of blended and online learning before or during COVID-19 lockdowns) (Al-Nofaie, 2020; Alghamdi and Ali, 2021; AlNajdi, 2015; Anas, 2020; Asiry, 2017; Castro et al., 2022; Khashaba et al., 2022). Further, these researches covered either all students (Khashaba et al., 2022) or those who belonged to a specific program, i.e., English (Al-Nofaie, 2020; Anas, 2020), dental (Asiry, 2017), nursing (Castro et al., 2022), pharmacy (Alghamdi and Ali, 2021). However, no studies have discovered the health sciences students' perception of blended and online learning, especially the public health and applied medical sciences students. Besides, previous literature mostly revealed the students' perception of blended or online learning during the COVID-19 lockdowns. Specifically, no studies have explored the students' perception of blended and online learning before, during, and after the COVID-19 lockdowns using a longitudinal study approach. Hence, this study intended to investigate students' perception of online and blended learning courses during all semesters (i.e., before, during, and after the COVID-19 lockdowns). Specifically, it focused on revealing the students' perception of online and blended learning in four key areas: satisfaction, convenience, engagement, and learning to examine the existing relationship between all students' perceptions in blended learning courses and their achievement in a course.

Thus, the “Computer Fundamentals” course offered to health information management and technology (HIMT) students at the College of Public Health (CPH) and cardiovascular & technology students of the College of Applied Medical Sciences (CAMS) of Imam Abdulrahman Bin Faisal University (IAU) were chosen to execute this research study. These programs use different levels of the blended learning approach. In a real scenario, Blended learning is offered to students 25% during the first and second semesters and 50% during the third semester. Surprisingly, during the fourth semester, the COVID-19 lockdowns disrupted the education system at IAU, and the course was offered through 75 % blended. The entire course was offered online during the fifth and sixth semesters. Further, the students were surveyed at the end of each semester to enhance the course quality.

Further, the research study establishes a connection between the students' views on online and blended learning and performance before and after the COVID-19 lockdowns. Knowing this connection will help policymakers of IAU and other HEIs understand the presence of any distinct impact between high and low achievers based on variables such as satisfaction, convenience, engagement, and achievement at various levels of blended or online courses (Owston and York, 2018). This research helps them design and support blended or online classes, usually including students of different levels. The findings of this study might also offer crucial information relevant for online distribution when establishing a blended course. It will enable them to adopt appropriate technology to satisfy their current net student generation’s expectations and requirements and improve the global education and learning environment.

### 1.1 Research questions

To be precise, this study was conducted with three-fold objectives, viz.

(i) To investigate the students' perception of satisfaction, convenience, engagement, and learning towards the blended and online course during various times (i.e., before, during, and after the COVID-19 lockdowns).
(ii) To determine whether the students' perception differs about various study factors such as ‘learning modes’, ‘examination modes’, and ‘student batches/across semesters’.
(iii) To examine whether the factors such as students' batch, mode of exams, and learning modes are associated with the student’s achievement.

## 2 Literature review

### 2.1 Blended learning

Blended learning is a blend of online and face-to-face learning, enhancing students' self-learning and learning performances. It is essential to improve the students' satisfaction with an effective blended learning system (Dinh et al., 2021). Developing robust student engagement in both face-to-face and online environments is essential for the delivery of active blended learning since student engagement is a requirement for positive learning (Lam et al., 2018).

Moreover, the blended approach delivers the convenience and flexibility of online courses with face-to-face interaction. Generally, students' satisfaction with blended learning is positive with convenience and regulation of the pace of learning. It is observed that most of the students enjoyed the convenience of the blended approach (Kenney, 2011). Blended learning results in improved students' learning and interaction, enhanced flexibility and access to content design and delivery, and more commitment to the learning process. It also has a significant favorable influence on academic achievement. It improves the students' achievement through the fusion of online and face-to-face learning (Najafi and Heidari, 2019).

### 2.2 Online learning

As online learning becomes more vital during the COVID-19 lockdowns, online learning is a valuable and practical tool for curriculum delivery during the new normal conditions. Satisfaction with online learning is a significant aspect of promoting successful educational processes for students, faculty, and HEIs. Students were satisfied with the flexibility and communication provided during online learning. Students' satisfaction is positively related to their engagement and academic performance (Elshami et al., 2021). Student engagement is another critical concern in online learning. Baloran et al. (2021) observed a high level of students' course satisfaction and engagement with online learning delivery. Rajabalee et al. (2020) showed a strong positive relationship between students' engagement and performance. However, engaging students in online learning is one of the instructors' challenges (Elshami et al., 2021).

Convenience is a vital feature to be measured in online learning, and it is essential to successfully implement online education in HEIs (Sulaiman, 2014). The benefits of online learning are mainly cost-effective, convenient, flexible, and self-learning. Online learning is convenient for students because they can access online materials (Almahasees et al., 2021). It also enhances easy access to effective learning, improving students' academic achievement. It significantly affects students' academic achievement (Mothibi, 2015).

## 3 Materials and methods

A longitudinal study design was adopted to examine the students' perception of online and blended learning courses before, during, and after the COVID-19 lockdowns. The authors used the convenient sampling approach to select those students who underwent the “Computer Fundamentals Course” offered at two colleges of IAU, i.e., CPH (N = 61) and CAMS (N = 69). Moreover, the authors included only those students studying the “Computer Fundamentals Course” taught by a single instructor during the three academic years (six semesters) (2018–2021) in this study. The total number of students in the selected two colleges is 130. The study period consists of 6 semesters, including 2018 Fall, 2019 Spring, 2019 Fall, 2020 Spring, 2020 Fall, and 2021 Spring. Out of these semesters, 2018 Fall, 2019 Spring, and 2019 Fall were before the COVID-19 lockdowns with the ‘blended 0.25’ mode of learning (75% face-to-face interaction [in-person attendance] and 25% online), ‘blended 0.25’ (75% face-to-face interaction and 25% online), ‘blended 0.50’ (50% face-to-face interaction and 50% online) learning mode, respectively. Further, 2020 Spring was conducted during the COVID-19 lockdowns with a ‘blended 0.75’ learning mode (75% online and 25% face-to-face interaction [in-person attendance]). The remaining 2020 Fall and 2021 Spring falls after the COVID-19 lockdowns with 100% online learning. The suitable examination mode, in-person or online, was conducted before, during, and after the COVID-19 lockdowns. The information about the mode of learning and examination for each batch of students is provided in Table 1.

**Table 1:** Mode of learning and exam mode for each batch during academic years 2018–2021.

Batch	Number of students	Mode of Learning	Mode of exam	Semester Status
2018 Fall	19	Blended 0.25 (25% online & 75% In-person class sections)	In-person attendance	Before COVID-19 (Normal)
2019 Spring	19	Blended 0.25 (25% online & 75% In-person class sections)		
2019 Fall	18	Blended 0.50 (50% online & 50% In-person class sections)		
2020 Spring	24	Blended 0.75 (75% online & 25% In-person class sections)	Online attendance	During COVID-19
2020 Fall	24	Online 100%	In-person attendance	After COVID-19 First Adaption
2021 Spring	26	Online 100%		After COVID-19 Second Adaption

The ethical approval for this study was obtained from the Institutional Review Board (IRB) of IAU, Saudi Arabia (IRB-2019-03-215). Further, all the participants completed the informed consent form before participation, and confidentiality and anonymity were assured before gathering the data from the participants. Based on the past literature, an online survey was developed and administered to the students of the selected two colleges before, during, and after the COVID-19 lockdowns. The survey consists of i) Demographic data covering the age, gender, and the details of the college, department, course, academic year, term, learning mode, final exam mode, blended level, and term status, ii) Statements covering various attributes of blended and online learning, and iii) Students' Preferences on Course format. The students' level of agreement towards each statement was recorded using a five-point Likert scale (“1-strongly disagree”, “2-disagree”, “3-neutral”, “4-agree”, “5-strongly agree”). Besides, this study also collected information about the course grades achieved by the students.

Descriptive statistics were applied to describe the demographic information of the samples and the responses to the survey items. Chi-square statistic was used to study the association between the various study factors and students' grades. The comparison of students' perception scores among the different study domains was performed using the Independent ‘t’ test and analysis of Variance (ANOVA). All statistical tests were carried out at 5% significance levels (P-value < 0.05). The entire analysis was carried out using IBM SPSS Statistics software version 25.

## 4 Results

### 4.1 Data description of study participants' characteristics

In this study, 130 students responded to the survey. While reviewing the results, 75% of the respondents were aged between 18 and 20 years. All the respondents were female. The data description regarding the course characteristics is presented below in Table 2.

**Table 2:** Data description of study participants regarding their course (N = 130).

Characteristics	Frequency (n)	Percentage (%)
Exam mode		
In-person attendance	106	81.5
Online attendance	24	18.5
Learning Mode		
Online 100%	50	38.5
Blended 0.75	24	18.5
Blended 0.50	18	13.8
Blended 0.25	38	29.2
Term		
Before COVID-19 (Normal)	56	43
During COVID-19	24	18.5
After COVID-19 First adaptation	24	18.5
After COVID-19 Second adaptation	26	20
College/Course		
College of Public Health/HIMT Program	61	46.9
College of Applied Medical Sciences/Cardiac Technology Program	69	53.1
Batch		
2018 Fall (Before COVID-19)	19	14.6
2019 Spring (Before COVID-19)	19	14.6
2019 Fall (Before COVID-19)	18	13.8
2020 Spring (During COVID-19)	24	18.5
2020 Fall (After COVID-19 First Adaption)	24	18.5
2021 Spring (After COVID-19 Second Adaption)	26	20

### 4.2 Description of perception items

Students' perception of blended and online learning is described in Tables 3 and 4. The results showed that 95.4% were overall satisfied with the course (Computer Fundamentals) (Table 3). Also, 60.8 % of the students preferred the blended 0.50-course format, with 50% online and the rest of it through direct face-to-face interactions (Table 4). For the items “If you had a choice between attending lectures face-to-face or accessing lectures online, which would you choose?” and “If you had a choice between participation in classroom discussion or online discussion, which would you choose?”, the majority of students responded as “Combination of Both” (Table 4).

**Table 3:** Students' perception of Blended and Online Learning from Fall 2018 to Spring 2021

S. No.	Items	Domain	Agree/Strongly Agree Frequency (n)	Percentage (%)
1	Overall, I am satisfied with this course.	Satisfaction	124	95.4
2	Given the opportunity, I would take another course in the future that has both online and face-to-face components	Satisfaction	112	86.2
3	When I encountered a problem with using the technologies in this course, the IAU technical support service helped me with my problem in a timely and effective manner.	Satisfaction	93	71.5
4	Accessing the course online lectures cost me so much money as compared to face-to-face lectures	Satisfaction	18	13.8
5	This course experience has improved my opportunity to access and use the class content.	Convenience	113	86.9
6	This course offered the convenience of not having to come to campus as often.	Convenience	101	77.7
7	This course allowed me to reduce my total travel time each week and related expenses.	Convenience	103	79.2
8	I feel connected with other students in this course	Convenience	110	84.6
9	I feel isolated during this course	Convenience	18	13.8
10	I have strong time management skills.	Convenience	71	54.6
11	The online and face-to-face course components of this course enhanced each other.	Engagement	110	84.6
12	The course blackboard site is well organized and easy to navigate.	Engagement	104	80
13	The web resources in this course are helpful.	Engagement	108	83.1
14	I am more engaged in this course.	Engagement	103	79.2
15	I am likely to ask questions in this course.	Engagement	89	68.5
16	I feel that the amount of my interaction with other students in this course increased.	Engagement	105	80.8
17	I feel that the quality of my interaction with other students in this course was better.	Engagement	99	76.2
18	I feel that the amount of my interaction with the instructor in this course increased.	Engagement	97	74.6
19	I feel that the quality of my interaction with the instructor in this course was better.	Engagement	95	73.1
20	I am overwhelmed with the information and resources in this course.	Engagement	59	45.4
21	I have trouble using the technologies in this course	Engagement	17	13.1
22	I feel more anxious in this course.	Engagement	27	20.8
23	This course required more time and effort	Engagement	44	33.8
24	This course has improved my understanding of key concepts	Learning	122	93.8
25	I am motivated to succeed.	Learning	100	76.9

**Table 4:** Students' perception towards course format preferences during the study period from Fall 2018 to Spring 2021

S. No.	Items	Responses	Frequency (n)	Percentage (%)
26	If the same course is being offered in different formats, which course format would you prefer?	Blended 0.25-course format *(meaning 25% face-to-face activities are replaced with online activities)*	4	3.1
		Blended 0.50-course format *(meaning 50% face-to-face activities are replaced with online activities)*	79	60.8
		Blended 0.75-course format *(meaning 75% face-to-face activities are replaced with online activities)*	16	12.3
		Entirely online course format *(with no face-to-face class time)*	31	23.8
27	If you had a choice between attending lectures face-to-face or accessing lectures online, which would you choose?	Accessing online downloadable videos of lectures	44	33.8
		A combination of both	86	66.2
28	If you had a choice between participation in classroom discussion or online discussion, which would you choose?	Class discussion	36	27.7
		Online discussion	40	30.8
		A combination of both	54	41.5

### 4.3 Perception score comparison among various study factors

Table 5 shows the results of the ANOVA depicting the students' perception of satisfaction, convenience, engagement, and learning concerning the various learning modes. It is found that there is a significant difference among the various modes of learning (Blended 0.25, 0.50, 0.75-course format, and 100% Online learning) concerning the students' perception of satisfaction, convenience, engagement, and learning (p < 0.05).

**Table 5:** Comparison of students' perception of various learning modes.

Domains of Students' Perception	Mode of Learning	Frequency (n)	Mean	Standard Deviation	P-value
Perception of Satisfaction	100% Online	50	20.7	1.8	0.014∗
	Blended 0.75 format	24	21.2	1.8	
	Blended 0.50 format	18	19.7	2.2	
	Blended 0.25 format	38	19.7	2.6	
Perception of convenience	100% Online	50	25.5	3.1	0.006∗
	Blended 0.75 format	24	25.5	3.7	
	Blended 0.50 format	18	22.4	2.8	
	Blended 0.25 format	38	24.6	3.4	
Perception of engagement	100% Online	50	56.3	5.8	0.002∗
	Blended 0.75 format	24	58.8	6.9	
	Blended 0.50 format	18	51.7	6.5	
	Blended 0.25 format	38	54.3	6.3	
Perception of learning	100% Online	50	9.0	1.2	0.007∗
	Blended 0.75 format	24	8.7	1.1	
	Blended 0.50 format	18	7.9	1.1	
	Blended 0.25 format	38	8.6	1.3	

∗ Significant at 0.05 level.

The Independent ‘t’ test demonstrated a significant difference between the examination modes (online and In-person mode) concerning students' satisfaction and engagement (p < 0.05). However, no significant difference was observed between the two examination modes regarding convenience and learning experienced by the students while taking these examinations (p > 0.05) (Table 6).

**Table 6:** Comparison of Students' perception of various modes of examination.

Domains of Students' Perception	Exam Mode	N	Mean	Std. Deviation	P-value
Perception of Satisfaction	In-person attendance	106	20.2	2.2	0.040∗
	Online attendance	24	21.2	1.8	
Perception of Convenience	In-person attendance	106	24.7	3.3	0.286
	Online attendance	24	25.5	3.7	
Perception of Engagement	In-person attendance	106	54.8	6.3	0.006∗
	Online attendance	24	58.8	6.9	
Perception of Learning	In-person attendance	106	8.7	1.2	0.916
	Online attendance	24	8.7	1.1	

∗ Significant at 0.05 level.

The results of the ANOVA test stated that there is a significant difference between various batches concerning the students' perception of convenience, engagement, and learning (p < 0.05), except for their satisfaction with the blended and online modes of learning (p > 0.05) (Table 7).

**Table 7:** Comparison of Students' Perception scores between the various batches.

Domains of Students' Perception	Batches	N	Mean	Std. Deviation	P-value
Perception of Satisfaction	2018 Fall	19	19.7	3.0	0.060
	2019 Spring	19	19.6	2.2	
	2019 Fall	18	19.7	2.2	
	2020 Spring	24	21.2	1.8	
	2020 Fall	24	20.8	1.6	
	2021 Spring	26	20.7	2.0	
Perception of Convenience	2018 Fall	19	24.1	3.4	0.021∗
	2019 Spring	19	25.2	3.5	
	2019 Fall	18	22.4	2.8	
	2020 Spring	24	25.5	3.7	
	2020 Fall	24	25.5	2.9	
	2021 Spring	26	25.5	3.3	
Perception of Engagement	2018 Fall	19	54.8	7.7	0.004∗
	2019 Spring	19	53.8	4.6	
	2019 Fall	18	51.7	6.5	
	2020 Spring	24	58.8	6.9	
	2020 Fall	24	57.7	5.7	
	2021 Spring	26	55.0	5.7	
Perception of Learning	2018 Fall	19	8.6	1.4	0.033∗
	2019 Spring	19	8.6	1.1	
	2019 Fall	18	7.9	1.1	
	2020 Spring	24	8.7	1.1	
	2020 Fall	24	9.0	1.3	
	2021 Spring	26	9.1	1.0	

∗ Significant at 0.05 level.

### 4.4 Association between various domains studied and grades of study participants

The proportion of grades among the different study factors is presented below in Table 8. The Chi-square test results revealed that the student’s grades were associated with the study factors such as batches/semesters, exam mode, and learning mode (p < 0.05). Among various batches (semesters), it is observed that 32–42% of the students scored A+ and A in the batches/semester, i.e., 2018 Fall, 2019 Spring, and 2019 Fall (before the COVID-19 lockdowns). In the 2020 Spring batch (during the COVID-19 lockdowns), 95.8% of the students scored A+, and 4.2% scored A. Further, no students had scored B+ and B. However, this trend has changed in the subsequent batches/semesters, i.e., 2020 Fall and 2021 Spring (after the COVID-19 lockdowns). In those semesters, most of them scored A.

**Table 8:** Chi-square statistic showing the association between study factors and the grades of students' participants.

Study Factors	Grades								P-value
	A+		A		B+		B		
	n	%	N	%	n	%	n	%	
Batch/Semester									<.001∗
2018 Fall	6	31.6	8	42.1	5	26.3	0	0	
2019 Spring	8	42.1	7	36.8	4	21.1	0	0	
2019 Fall	6	33.3	7	38.9	4	22.2	1	5.6	
2020 Spring	23	95.8	1	4.2	0	0	0	0	
2020 Fall	6	25	11	45.8	6	25	1	4.2	
2021 Spring	7	26.9	10	38.5	9	34.6	0	0	
Exam mode									<.001∗
In-person attendance	33	31.1	43	40.6	28	26.4	2	1.9	
Online attendance	23	95.8	1	4.2	0	0	0	0	
Learning mode									<.001∗
100% Online (2020 Fall)	6	25	11	45.8	6	25	1	4.2	
100% Online (2021 Spring)	7	26.9	10	38.5	9	34.6	0	0	
Blended 0.75 (2020 Spring)	23	95.8	1	4.2	0	0	0	0	
Blended 0.50 (2019 Fall)	6	33.3	7	38.9	4	22.2	1	5.6	
Blended 0.25 (2018 Fall)	6	31.6	8	42.1	5	26.3	0	0	
Blended 0.25 (2019 Spring)	8	42.1	7	36.8	4	21.1	0	0	

∗ Significant at 0.01 level.

Concerning the exam mode, the students wrote their examination before COVID-19 (i.e., 2018 Fall, 2019 Spring, and 2019 Fall) and after COVID-19 (i.e., first adaption and second adaptation) through “In-Person.” In contrast, the students wrote their examinations online during COVID-19 (i.e., 2020 Spring). Notably, 95.8% of students who wrote their final exam online during the COVID-19 lockdowns scored an A+ grade, and 4.2% achieved an A grade. At the same time, 40.6% of students who wrote the examination ‘in-person’ scored an A grade, and 31.1% scored an A+ grade.

Regarding the learning mode, 42.1% of students scored A grade, and 31.6% scored A+ grade through Blended (25% Online learning) during the 2018 Fall (Before COVID-19). During the 2019 Spring (i.e., Before COVID-19), the percentage of students who scored A and A+ grades through Blended (25% Online learning) was observed as 36.8% and 42.1%, respectively. 38.9% of students achieved A-grade, and 33.3% scored A+ grade through Blended (50% Online learning) during the 2019 Fall. Notably, a high percentage (95.8%) of students scored A+ grade through Blended (75% Online learning) during COVID-19. After COVID-19, the students used 100% online learning during the first and second adaptation. Most of them scored A grade during the first adaptation (45.8%) and second adaptation (38.5%), respectively.

## 5 Discussion

This study revealed the students' perception of blended and online learning at various times spread over six semesters. Further, this study brings out students' perceptions regarding satisfaction, convenience, engagement, and learning that vary across different learning modes. Also, it reveals the association between three study factors such as learning mode, mode of exam, students' batch/semester, and the student’s achievement. This study is more relevant in the current scenario, as COVID-19 highly influenced higher education’s teaching and learning system.

### 5.1 Students' perceptions concerning satisfaction, convenience, engagement, and learning

While reviewing the results, 95.4% of students were satisfied with the course entitled “Computer Fundamentals,” which was explored in this research study for six academic semesters. Among the variables studied, most students (93.8%) perceived that the course had improved their understanding of critical concepts. On the other hand, a few students (13.8%) felt isolated during the course (13.8%) when offered through an online or blended learning model. Notably, it is a positive finding as only a few students felt isolated during the course. In conformance with our results, an earlier study also stated that students described feeling isolated from their teachers and classmates when courses were delivered online or blended mode (Boling et al., 2012). A feeling of isolation is observed as one of the significant disadvantages of online learning, resulting from the lack of interaction with peers and faculty (Coman et al., 2020). Online students may feel isolated due to reduced or lack of interaction (Dixson, 2015; Kwary and Fauzie, 2018). Hence, it is essential to consider learning as a social process while developing online learning experiences. In addition, Banna et al. (2015) highlighted that engagement is the crucial solution to the issue of students' isolation in online learning. Previous studies declare the significance of student engagement in online learning as it is evident for their cognitive development and knowledge enrichment, which lead to student success (Banna et al., 2015; Britt, 2015; Meyer, 2014). Therefore, engagement strategies focusing on delivering positive student experiences such as collaborative group work, presentations, discussions, case studies, and sharing resources can be implemented while designing the online courses (Martin and Bolliger, 2018). Further, 13.8% of participants felt accessing online lectures is costlier than face-to-face lectures. Contrarily, Dhawan (2020) stated that the online learning mode is easily accessible and can even reach rural and remote areas. It is considered a relatively cheaper mode of education because of the lower cost of transportation, accommodation, and the overall cost of institution-based learning. However, a recent study stated that insufficient broadband infrastructure is a critical obstacle to the online learning delivery in rural locations. Strategies to progress policies and improve technology must consider geographical differences to warrant education equity (Graves et al., 2021).

### 5.2 Students' perception of course format preferences

Concerning the course format preferences, this study demonstrated that over 60% of participating students preferred courses to be offered through a Blended 0.50-course format. Most students (66.2%) liked the combination of face-to-face and online lectures. A considerable number of students (41.5%) were interested in participating in both classroom and online discussions. Contrary to these results, Kemp and Grieve (2014) observed that the students favoured finishing their events face-to-face than online, and those stated their solid preference for class discussions to be conducted face-to-face.

### 5.3 Students' perception of various learning modes

While comparing the learning modes, the student’s perception of satisfaction, convenience, engagement, and learning on those courses offered through the Blended0.50 learning format (50% online) during the 2019 fall is rated as ‘low.’ The reasons for this low score during this semester are unclear, and further qualitative studies must be conducted among instructors and students. Moreover, the highest perception score for satisfaction and engagement was observed towards the Blended 0.75 learning format (75% online learning) that was implemented during the COVID-19 lockdowns. Conversely, the perception score of learning was found to be high when the course was offered through a 100% online learning format (After COVID-19). Notably, the students perceived their convenience equally with both Blended (75% online learning) and 100% online learning formats. Even though this research reveals that the Blended 0.75 learning format had higher perception scores on most of the factors explored, most students responded that they preferred the Blended 0.50 learning format among all the course formats studied. Hence, the lectures and discussions can be conducted online and face-to-face mode in the future (Owston et al., 2013). This observation is also in line with a previous study by Herbert et al. (2017). Another recent Saudi Arabian study also highlighted the importance of online learning in higher education (Walabe and Luppicini, 2020). Moreover, uniform rules and regulations are necessary for delivering blended learning systems to sustain the quality of higher education during lockdowns.

Besides, a significant difference was observed between the various learning modes (25%, 50%, 75% of Blended learning, and 100% of Online learning) concerning the students' perception of satisfaction, convenience, engagement, and learning. An earlier study also indicated that students' perceptions differ concerning various learning modes and face-to-face learning is always perceived higher than online learning in terms of social presence, social interaction, and satisfaction (Bali and Liu, 2018).

### 5.4 Students' perception over various examination modes

Regarding the examination modes, the students' perception score of all domains except learning among those who appeared through the online examination mode is higher than those through the “In-person” mode. However, the perception score for learning among students who appeared through online and In-person modes are similar. In line with these findings, Ali et al. (2021) observed that Saudi pharmacy students, during the COVID-19 lockdown adopted technology for their e-learning resources, online lectures, online learning, and online examinations. In this study, the students attended their exams online only during the COVID-19 lockdowns (2020 Spring). Further, the students experienced easy access to technology which might enhance their online learning during the pandemic. Besides, this study observed a significant difference between the examination modes (online and In-person attendance mode) regarding the students' satisfaction and engagement. However, no significant difference was observed between the two examination modes regarding convenience, and learning indicated that the students were comfortable taking exams both online and in-person mode. In conformance with our findings, a study by Alghamdi and Ali (2021) concluded that Saudi pharmacy students perceived that the online examinations were stress-free and felt comfortable in their homes. Also, they could highly focus on online examinations compared to on-campus ones. Despite this, it is recommended that future studies discover the influence of online examination on student learning using proper methods rather than students' viewpoints. Besides, both faculty and students felt that online exams give more chances for cheating than those in-person exams (Holden et al., 2021). King and Case (2014) observed higher cheating rates in online and in-person exams. Furthermore, most students often commit some academic dishonesty, such as cheating and plagiarism, to achieve higher grades than they are capable of (Hosny and Fatima, 2014). Plagiarism is the central issue in online assessment since the internet provides massive information that may be misused using modern technology. It is essential to prevent and detect plagiarism in online assessments. Submitted information is screened for plagiarism and tracked down the originality using software (Wannige et al., 2008). A recent study stated that software tools could be incorporated into online exams to detect plagiarism (Sabrina et al., 2022). Hence, appropriate strategies are required to reduce academic dishonesty in higher education while conducting online exams.

Besides, both online and in-person assessment format differs from each other. In-person assessment is widely used, and its tools include multiple-choice questions (MCQs), true/false tests, short answers, and essays (Dikli, 2003). It emphasizes a restricted count of high-stakes student exams that are directly supervised by instructors (proctored exams). Such supervised exams reduce cheating (Fynn and Mashile, 2022). On the other hand, the shift towards online learning during 2020 made the use of invigilated in-person exams to online assessments. Online assessment is designed to develop or measure cognitive levels, including MCQs, true/false tests, matching, and short responses. It can be proctored or unproctored (Mate and Weidenhofer, 2022). During the online assessment, instructors and students are involved in a series of micro assessments focusing on supporting the learner through the several skills and knowledge systems required by the curriculum during the teaching period. Online assessment relies on the technology infrastructure and students' access to the digital devices. Instructors reported that cheating and poor participation are the encounters of online assessment (Fynn and Mashile, 2022). This condition can be considered when analysing the achievement by online and in-person exams.

### 5.5 Variation in students' perception of satisfaction, convenience, engagement, and learning concerning batches

While reviewing the students' perception among various batches (i.e., across different semesters studied), the lowest mean score of convenience, engagement and learning was observed during the 2019 Fall (i.e., Before COVID-19). For satisfaction, the lowest mean score was observed during the 2019 Spring (i.e., Before COVID-19). Subsequently, there was a higher perception score for all the domains except learning during the 2020 Spring (i.e., During COVID-19). The instructor introduced the blended with 75% Online course during the 2020 Spring due to the COVID-19 lockdowns. This observation could be because of the higher proportion of online learning held during the COVID-19 pandemic (Blended 75% Online learning), as the students are engaged more online than in face-to-face presentations. The course was very well designed; hence the COVID-19 pandemic did not affect the perception scores except in learning. Moreover, with the Blended 0.75 learning format, students get chances to meet with their friends and teachers in the university, and this format makes students safely and remotely attend the course. This condition may have helped them better perceive convenience, engagement, and satisfaction. Hence, it is good to be a blended 0.75 learning format as it includes classroom activities and presentations along with online classes and activities. According to this study, a blended 0.75 has a higher perception score which is in line with an earlier study by Owston et al. (2019). Previous studies also mentioned that high satisfaction with the blended learning method (Awamleh, 2020; Rienties et al., 2015). However, it is noteworthy that during the 2020 Spring (i.e., During COVID-19), the students did not perceive the learning with the highest mean score compared to other domains. This finding might be due to the increase in the proportion of online components in the Blended (75% online) learning mode during the COVID-19 compared to other blending learning formats adopted during other semesters (i.e., 2018 Fall, 2019 Spring, and 2019 Fall). Conversely, the students of 2020 Fall (After COVID-19 First adaption) and 2020 Spring (After COVID-19 Second adaption) perceived the learning through 100% Online learning mode with the highest mean score. Similar to this finding, O'Dea and Zhou (2022) stated that several HEIs in UK considered adopting blended learning in the post-COVID-19 era due to the advantages of online learning like accessibility, flexibility, and self-paced learning. Our findings also indicate that students' perception scores concerning satisfaction, convenience, and learning experienced after blended and online learning are found to be higher during the first and second adaptation following COVID-19 lockdowns. It can be inferred from the last two batches (i.e., 2020 Fall and 2021 Spring), that the students turned into expertise in using the new online learning methods and managing technology-based learning through online workshops/training sessions. This state helped to increase the perception of learning during these two semesters. Some students favour online classes because they save their time and money by not driving to the university. In this study, 79.2% of the study participants prefer online classes due to this factor. Likewise, Muthuprasad et al. (2021) observed that 70% of the students preferred to choose online classes to deal with the curriculum during the COVID-19 pandemic. Those students felt that convenience and flexibility were the attractive features of online classes. In general, student engagement is high among the blended learning group (Lima et al., 2021). However, in this study, while observing students' perception scores during the 2021 Spring, their engagement was less since COVID-19 restrictions are still prevailing, which may have influenced them psychologically.

### 5.6 Association between various domains studied and students' achievement

In this study, students' achievement (i.e., grades) showed a significant association with the factors such as student batches, learning modes, and examination modes. During the 2020 Spring, the achievement level was high than in other semesters. Similarly, those who appeared in exams online showed a high achievement than those who appeared in In-person mode. Also, the Blended 0.75 group had a high proportion of achievement than the other three groups. According to these findings, several researchers found that the students' achievement depends on the mode of teaching and instructional approach adopted (Bernard et al., 2014; Means et al., 2013; Zhao et al., 2005). Specifically, blended learning effectively improves students' achievement in computer studies (Ezeanyika and Okigbo, 2021).

This study is only limited to health science students of a single public university (IAU); hence, it is hard to generalize its findings in the Saudi Arabian context. Before the generalization of these findings, further research is warranted to cover the students of all disciplines of both public and private Saudi universities to represent larger sample size. Variations in students' achievement regarding the students' batches, learning mode, and examination mode can be explored in future studies. The influence of various teaching methods adopted in blended and online learning on students' achievement can also be discovered. As health science students are actively participate in direct patient care, effective teaching practices tailored to them would facilitate their blended and online learning process and improve their performance. From an implementation perspective, Turnball et al. (2021) stated that observations, interviews, and proctored examinations require a synchronous approach in an online learning environment. Hence, such an approach can be considered while conducting examinations and reveal the students' perception of examinations in future studies.

## 6 Conclusion

This is the first study to reveal the health science students' perception of the various blended and online learning modes implemented during the Normal and New conditions in a Saudi HEI. Over 95% of students are satisfied with the course offered through various blended learning formats. 94% expressed that such blended & online education has improved their understanding of key concepts; however, students feel isolated when the course is offered either entirely online or blended learning model. Further, this study uncovers students' satisfaction, convenience, engagement, and learning gained through blended and online learning formats at three intervals, i.e., before, during, and after the COVID-19 lockdowns. The lowest mean students' perception score for convenience, engagement, and learning was witnessed when blended, and online learning formats were practiced before COVID-19 (2019 Fall). However, such a scenario has changed during the lockdowns, and students have started appreciating the value of blended and online learning. Further, increased students' satisfaction, convenience, engagement, and enhanced learning occurs during the adaptation period following the lockdowns. Since then, the course has been 100% online; students rated it high and are accustomed to the blended and online system in the new normal environment.

The majority of the students preferred a Blended 0.50-course format as their choice for better learning. However, the Blended 0.75 online learning model is highly rated by the students, with a higher score for satisfaction and engagement than other learning formats. Moreover, following the COVID-19 adaption, the student’s perception of learning through a 100% online learning format is relatively high. Thus, it is concluded that students felt equally convenient with Blended 0.75 and 100% online learning formats, and uniform guidelines for universities delivering blended learning system is warranted to improve and sustain the quality of teaching and learning process in the event of a future pandemic.

The study also reveals that the examination mode impacts students' perception. The students' satisfaction, convenience, and engagement while taking exams through online attendance mode is higher than those through “In-person” attendance mode. This study also demonstrated an association between students' achievement (i.e., grades) and learning mode. The blended 0.75 group shows a higher achievement than the other three groups. Likewise, those students who appeared in their exams online showed a higher achievement than those who appeared in In-person attendance mode. Students might commit plagiarism to obtain higher grades in online exams, thereby software aids to prevent and detect it. Effective measures support policy planners of HEIs in controlling academic dishonesty in online exams. Also, the nature of online and in-person assessment formats differs from each other. However, exploring the reasons behind such higher achievement trends is beyond this study’s scope, and further research is warranted. Further, the findings of this research study will help educational policy planners and curriculum developers to choose appropriate learning and examination modes while designing courses. Also, the students should be exposed to workshops/training sessions on online learning platforms, which would help them enrich their readiness for online learning during upcoming disasters.

## Declarations

### Author contribution statement

Nouf Al-Kahtani, D.Sc: Conceived and designed the experiments; Performed the experiments; Contributed reagents, materials, analysis tools or data; Wrote the paper.

Abdullah Almurayh, PhD; Hend Alkahtani, PhD; Duaa Aljabri, PhD: Performed the experiments.

Arun Vijay Subbarayalu, PhD: Analyzed and interpreted the data; Wrote the paper.

Tunny Sebastian, PhD: Analyzed and interpreted the data; Contributed reagents, materials, analysis tools or data.

### Funding statement

This research did not receive any specific grant from funding agencies in the public, commercial, or not-for-profit sectors.

### Data availability statement

Data will be made available on request.

### Declaration of interest’s statement

The authors declare no conflict of interest.

### Additional information

No additional information is available for this paper.

## Appendix A Supplementary data

Online _spl_ Blended survery tool Online _spl_ Blended survery tool
