# Supplementary material for: Sustaining blended and online learning during the normal and new normal conditions in a Saudi higher education institution: health science students' perspectives
Source: Heliyon. 2022 Oct 4;8(10):e10898. doi: 10.1016/j.heliyon.2022.e10898 (PMC9562221; doi:10.1016/j.heliyon.2022.e10898)
Supplement: Online _spl_ Blended survery tool [file mmc1.docx]

**ONLINE AND BLENDING LEARNING SURVEY FOR STUDENTS**

**I. DEMOGRAPHICAL DATA**

**Age: ___________** (in years)

**Gender:** Male Female

**College:** College of Applied Medical Sciences College of Public Health

**Department:** Health Information Management (HIMT) Cardiovascular Technology (CT)

**Course Title:** _____________________

**Academic Year:** _______________

**Term:** 2018 Fall 2019 Spring 2019 Fall 2020 Spring 2020 Fall 2021 Spring

**Final Exam Mode:** In Person Online

**Learning Mode:** Blended Online

**Level of Blended:** 25% 50% 75% 100%

**Term Status:** Before COVID-19 (Normal) During COVID-19

After COVID-19 First Adaption After COVID-19 First Adaption

**II. STUDENTS’ PERCEPTION TOWARDS VARIOUS ATTRIBUTES OF BLENDED AND ONLINE LEARNING**

***Please select any one response to each question. Be assured that your responses will be kept confidential***

| **#** | **Statements** | **Domain** | **Strongly Agree**  **(5)** | **Agree**  **(4)** | **Neutral(3)** | **Disagree(2)** | **Strongly Disagree(1)** |
| --- | --- | --- | --- | --- | --- | --- | --- |
| 1 | Overall, I am satisfied with this course. | Satisfaction |  |  |  |  |  |
| 2 | Given the opportunity I would take another course in the future that has both online and face-to-face components. | Satisfaction |  |  |  |  |  |
| 3 | When I encounter a problem with the use of the technologies in this course, the IAU technical support service helped me with my problem in a timely and effective manner. | Satisfaction |  |  |  |  |  |
| 4 | Accessing the course online lectures cost me so much money as compared to face-to-face lectures | Satisfaction |  |  |  |  |  |
| 5 | This course experience has improved my opportunity to access and use the class content. | Convenience |  |  |  |  |  |
| 6 | This course offered the convenience of not having to come to campus as often. | Convenience |  |  |  |  |  |
| 7 | This course allowed me to reduce my total travel time each week and related expenses. | Convenience |  |  |  |  |  |
| 8 | I feel connected to other students in this course. | Convenience |  |  |  |  |  |
| 9 | I feel isolated during this course. | Convenience |  |  |  |  |  |
| 10 | I have strong time management skills. | Convenience |  |  |  |  |  |
| 11 | The online and face-to-face components of this course enhanced each other. | Engagement |  |  |  |  |  |
| 12 | The course blackboard site is well organized and easy to navigate. | Engagement |  |  |  |  |  |
| 13 | The web resources in this course are helpful. | Engagement |  |  |  |  |  |
| 14 | I am more engaged in this course. | Engagement |  |  |  |  |  |
| 15 | I am more likely to ask question in this course. | Engagement |  |  |  |  |  |
| 16 | I feel that the amount of my interaction with other students in this course increased. | Engagement |  |  |  |  |  |
| 17 | I feel that the quality of my interaction with other students in this course was better. | Engagement |  |  |  |  |  |
| 18 | I feel the amount of my interaction with instructor in this course. | Engagement |  |  |  |  |  |
| 19 | I feel the quality of my interaction with instructor in this course was better. | Engagement |  |  |  |  |  |
| 20 | I am overwhelmed with the information and resources in this course. | Engagement |  |  |  |  |  |
| 21 | I have trouble using the technologies in this course. | Engagement |  |  |  |  |  |
| 22 | I feel more anxious in this course. | Engagement |  |  |  |  |  |
| 23 | This course required more time and effort. | Engagement |  |  |  |  |  |
| 24 | This course has improved my understanding of key concepts. | Learning |  |  |  |  |  |
| 25 | I am motivated to succeed. | Learning |  |  |  |  |  |

**III. STUDENTS’ PREFERENCES ON COURSE FORMAT**

***Please select any one response to each question. Be assured that your responses will be kept confidential***

| **#** | **Statements** | **Domain** | **Responses** |
| --- | --- | --- | --- |
| 26 | If the same course is being offered in different formats, which course format would you prefer? | Satisfaction | Entirely face-to-face course format  25% Blended course format (meaning 25% face-to-face activities are replaced with online activities)  50% Blended course format (meaning 50% face-to-face activities are replaced with online activities)  75% Blended course format (meaning 75% face-to-face activities are replaced with online activities)  Entirely online course format (with no face-to-face class time) |
| 27 | If you had a choice between attending lectures face-to-face or accessing lectures online which would you choose? | Engagement | Attending lectures face-to-face  Accessing online downloadable videos of lectures  A combination of both |
| 28 | If you had a choice between participation in classroom discussion or online discussion, which would you choose? | Engagement | Class discussion  Online discussion  Combination of both A combination of both |

***Thank you***
